# Author Correction: Analysis of SARS-CoV-2 mutations in the United States suggests presence of four substrains and novel variants

**DOI:** 10.1038/s42003-021-01867-y

**Published:** 2021-03-03

**Authors:** Rui Wang, Jiahui Chen, Kaifu Gao, Yuta Hozumi, Changchuan Yin, Guo-Wei Wei

**Affiliations:** 1 Department of Mathematics, Michigan State University, East Lansing, MI 48824 USA; 2 Department of Mathematics, Statistics, and Computer Science, University of Illinois at Chicago, Chicago, IL 60607 USA; 3 Department of Electrical and Computer Engineering, Michigan State University, East Lansing, MI 48824 USA; 4 Department of Biochemistry and Molecular Biology, Michigan State University, East Lansing, MI 48824 USA

**Keywords:** SARS-CoV-2, Genome informatics, Viral infection

Correction to: *Communications Biology* 10.1038/s42003-021-01754-6, published online 15 February 2021.

In the original version of the Article, the final reference to SARS-CoV-2 in the abstract was incorrectly spelled as SASR-CoV-2. The error has been corrected in the PDF and HTML versions of the Article.

